# What is the hype on #MedicinalCannabis in the United States? A content analysis of medicinal cannabis tweets

**DOI:** 10.1111/dar.13618

**Published:** 2023-02-21

**Authors:** Carmen C. W. Lim, Tianze Sun, Coral Gartner, Jason Connor, Marco Fahmi, Wayne Hall, Sam Hames, Daniel Stjepanović, Gary Chan, Janni Leung

**Affiliations:** ^1^ National Centre for Youth Substance Use Research The University of Queensland Brisbane Australia; ^2^ School of Psychology The University of Queensland Brisbane Australia; ^3^ NHMRC Centre of Research Excellence on Achieving the Tobacco Endgame, School of Public Health The University of Queensland Brisbane Australia; ^4^ Discipline of Psychiatry The University of Queensland Brisbane Australia; ^5^ School of Languages and Cultures The University of Queensland Brisbane Australia; ^6^ Digital Observatory Queensland University of Technology Brisbane Australia

**Keywords:** medicinal cannabis, medicinal marijuana, Twitter, United States

## Abstract

**Introduction:**

Medicinal cannabis is now legal in 44 US jurisdictions. Between 2020 and 2021 alone, four US jurisdictions legalised medicinal cannabis. The aim of this study is to identify themes in medicinal cannabis tweets from US jurisdictions with different legal statuses of cannabis from January to June 2021.

**Methods:**

A total of 25,099 historical tweets from 51 US jurisdictions were collected using Python. Content analysis was performed on a random sample of tweets accounting for the population size of each US jurisdictions (*n* = 750). Results were presented separately by tweets posted from jurisdictions where all cannabis use (non‐medicinal and medicinal) is ‘fully legalised’, ‘illegal’ and legal for ‘medical‐only’ use.

**Results:**

Four themes were identified: ‘Policy’, ‘Therapeutic value’, ‘Sales and industry opportunities’ and ‘Adverse effects’. Most of the tweets were posted by the public. The most common theme was related to ‘Policy’ (32.5%–61.5% of the tweets). Tweets on ‘Therapeutic value’ were prevalent in all jurisdictions and accounted for 23.8%–32.1% of the tweets. Sales and promotional activities were prominent even in illegal jurisdictions (12.1%–26.5% of the tweets). Fewer than 10% of tweets were about intoxication and withdrawal symptoms.

**Discussion and Conclusion:**

This study has explored if content themes of medicinal cannabis tweets differed by cannabis legal status. Most tweets were pro‐cannabis and they were related to policy, therapeutic value, and sales and industry opportunities. Tweets on unsubstantiated health claims, adverse effects and crime warrants continued surveillance as these conversations could allow us to estimate cannabis‐related harms to inform health surveillance.

**Key Points**

- Content analysis was used to examine themes related to medicinal cannabis tweets.
- Majority of the tweets were mainly pro‐cannabis and posted by the public.
- The most common theme was policy and was prevalent in illegal jurisdictions.
- Sales and promotional activities tweets were prominent even in illegal jurisdictions.
- Few tweets were about intoxication and withdrawal symptoms.

## 1 INTRODUCTION

There has been a growing use of cannabis to treat medical conditions such as chronic pain [1, 2], insomnia [3] and mental health symptoms [4] despite limited evidence on the safety and effectiveness of cannabinoids for these conditions. Around 27% of survey respondents from Canada and the United States reported using cannabis to manage these symptoms and the rates were especially high among young adults [5].

As of 2021, US federal law classifies cannabis as a Schedule 1 drug of no medical value and a high potential for abuse [6]. Medicinal cannabis policies in each US jurisdiction range from a restrictive approach that only permits the use of medicinal cannabis for a few qualifying medical conditions to liberal approaches that have legalised cannabis for medical and non‐medical use. Advocates of legalising adult cannabis use argue that cannabis has medical benefits [7] and other economic benefits such as increase in sales tax revenue, employment opportunities and lower law enforcement costs [8]. However, the concerns on increased used among young people, normalisation and exacerbation of the burden of mental illness [9] remain top concern should US legalise cannabis federally for non‐medical use. Since public opinion has greatly shifted in favour of legalisation [9], understanding public opinion and intentions are imperative for future cannabis legislation.

Routine surveillance through national surveys that examined the impact of different cannabis laws on public attitudes [10] often lag in data collection and reporting. Twitter is a micro‐blogging and real‐time communication social media platform used by millions of people to share information and form opinions on cannabis‐related topics. Twitter has been used by researchers to examine public opinion and behavioural intentions towards cannabis between 2014 and 2020 [11, 12, 13, 14, 15, 16, 17, 18]. In these studies, the volume of tweets was greater in jurisdictions that have legalised cannabis for non‐medicinal and/or medicinal use [13, 14, 15, 16] and most tweets were pro‐cannabis [12, 13]. Cannabis‐related discussions in jurisdictions with liberal cannabis policies also differed significantly in their content from those in jurisdictions with more restrictive policies [13]. Between 2020 and 2021, four US jurisdictions (Alabama, Louisiana, South Dakota and Virginia) greatly expanded access to or have legalised medicinal cannabis.

This study aims to identify emerging themes in medicinal cannabis tweets from US jurisdictions with different legal status.

## 2 METHODS

### 2.1 Data access and cleaning

The search strategy for medicinal cannabis tweets consisted of: (i) medicinal cannabis and its related terms (e.g., medicinal marijuana); and (ii) cannabis and its related terms referencing one or more common conditions for which medicinal cannabis is used (e.g., anxiety) (see Table S1, Supporting Information). In total, 121,308 historical tweets posted between 1 January 2021 and 30 June 2021 were collected using the ‘twarc2’ python library [19]. The country and the jurisdiction of each tweet was coded based on user‐specified location. Tweets with a missing location or posted outside of the United States were excluded. Duplicate tweets and retweets were removed, leaving a final sample of 25,099 unique tweets. The 50 US states and Washington DC were categorised into three groups according to their legal status as of 30 June 2021: (i) ‘fully legalised’ for both adult non‐medicinal and medicinal use; (ii) ‘medical only’; (iii) ‘illegal’ for both adult non‐medicinal and medicinal use (see Table S2). The grouping of legal status was consistent with a previous study examining cannabis‐related tweets from 2017 to 2019 [18] and the International Cannabis Policy Study [20].

### 2.2 Content analysis

A content analysis was conducted on a random sample of tweets using the qualitative methodology laid out in prior research analysing the themes of cannabis related tweets [17] and YouTube videos [21, 22]. Two researchers (Carmen Lim and Tianze Sun) independently read through the tweets and took initial notes to get familiar with the dataset. Short labels (codes) were derived to describe the content of 100 tweets. Patterns was identified among the codes to generate the preliminary themes and sub‐themes. Both researchers met to compare and harmonise the coding. The process was repeated on another set of 100 tweets. Each tweet can have multiple themes or sub‐themes. Data saturation was reached (i.e., no new themes emerged) and the codebook was deemed operational with clear definitions for themes and sub‐themes after 200 tweets. Fifty tweets per jurisdiction type (fully legalised, medical only and illegal) were additionally coded to confirm that there were indeed no new emerging themes. The researchers analysed 250 tweets per jurisdiction type (750 tweets in total). To ensure the representation of tweets across the United States, a set of weights were created for each jurisdiction where larger weights represent more populous states like California and vice versa. Weights were applied during the selection of tweets for each jurisdiction type (see Table S2). The inter‐rater reliability (Kappa coefficient = 0.8), indicated a substantial agreement [23] between both researchers. Any discrepancies in coding were resolved through consensus. The themes were tabulated and analysed.

## 3 RESULTS

Tweets were mainly posted by the public (52.4%), cannabis industry (17.1%) and advocates (9.6%). Only 1.2% were suspected to be ‘bot or spam’ accounts (see Table S3). Table 1 shows the themes derived from content analysis based on a random sample of 750 tweets from the full dataset (*n* = 25,099). The following themes were identified from all jurisdictions: ‘Policy’, ‘Sales and industry opportunities’, ‘Therapeutic value’ and ‘Adverse effects’. Approximately 8% (*n* = 58) of the tweets featured more than one theme. Despite the similarities in the themes found, the proportion of tweets varied by jurisdiction type.

**TABLE 1:** Frequencies and proportion of themes and sub‐themes associated with medicinal cannabis tweets (*n* = 750).

Themes and sub‐themes^a^	Definition	Examples	Fully legalised (*n* = 250)		Medical only (*n* = 250)		Illegal (*n* = 250)	
			*n*	%	*n*	%	*n*	%
**A. Policy** ^b^			87	32.5	125	46.8	168	61.5
(i) Support legalisation^c^	Individual or organisation expressing support towards medicinal and/or recreational cannabis legalisation	‘We urgently need to legalise cannabis. I would grow cannabis for my [family] if they were dying or needed it'.	35	11.3	43	13.9	97	30.4
(ii) Oppose legalisation^c^	Individual or organisation opposing medicinal and/or recreational cannabis legalisation	‘The [state organisation] has sent the constitutional amendment to forever ban medical marijuana'.	5	1.6	16	5.2	49	15.4
(iii) Other law‐related issue^c^	Law reforms, current policy, crime and illicit market‐related	‘Medical cannabis costs 350 an ounce and in black market only paid 210. The 40% or so state tax is ridiculous ….'	27	8.7	39	12.6	29	9.1
(iv) Medicinal cannabis program^c^	Discussion related to medicinal cannabis program (e.g., benefits of enrolling in a medicinal cannabis program)	‘ A bid to expand [state] medical marijuana program to allow patients to smoke cannabis sailed through its first review '.	21	6.8	28	9.0	4	1.3
(v) Inaccessibility of medicinal cannabis^c^	Inaccessibility of medicinal cannabis due to high state tax, high cost of medicinal cannabis, insurance cover, moving out of state to access cannabis	‘I have a [sick] daughter and I'm begging for our legislature to get to moving and implement a medical marijuana program yesterday. Shame on our Supreme Court'.	9	2.9	19	6.1	23	7.2
**B. Sales and industry opportunities** ^b^			71	26.5	53	19.9	33	12.1
(i) Promotional activities and discount codes^c^	Promotions and advertisement on cannabis products	‘Use coupon code relax—1ML CARTS—$29'.	25	8.1	30	9.7	18	5.6
(ii) Therapeutic claims^c^	Therapeutic claims of the specific product made by the vendors on medicinal cannabis for chronic medical conditions such as pain management, anxiety and epilepsy	‘Our [product] contains a [type] extract that can assist our furry friends in relieving joint pains, easing separation anxiety, promoting coat health, and much more'.	27	8.7	6	1.9	11	3.4
(iii) Industry‐related profits and business expansion^c^	Cannabis industry profits, share markets and business expansion	‘Roughly [*n*] years after the first medical marijuana dispensary opened in [state], the market has seen the sale of more than [*x*] pounds valued at [*x*] million.'	23	7.4	18	5.8	7	2.2
**C. Therapeutic value** ^b^			86	32.1	67	25.1	65	23.8
(i) User testimonial^c^	The use of medicinal cannabis to treat chronic medical conditions	‘I use cannabis to replace anxiety pills and pain pills'.	80	25.9	61	19.7	58	18.2
(ii) Product and dosage recommendation^c^	Recommendations (e.g., dosage, products) made to other Twitter users based on own experiences with medicinal cannabis	‘I manage my anxiety with [product]—here is my go to for my daily doses—[product]–[flavour] [*x*] ml − [*x*] mg'.	20	6.5	18	5.8	5	1.6
(iii) Research and success stories^c^	Tweets referencing research articles on medicinal cannabis to support their argument or other success stories posted by medicinal cannabis patients	‘Study: nearly half of medical cannabis users cease using opioids for pain after 12 months'.	13	4.2	10	3.2	11	3.4
**D. Adverse effects** ^b^			24	9.0	22	8.2	7	2.6
(i) Intoxication^c^	User‐reported symptoms associated with medicinal cannabis use such as being stoned, euphoric and giggly etc.	‘I'm stoned'.	7	2.3	9	2.9	1	0.3
(ii) Withdrawal symptoms^c^	Cannabis withdrawal symptoms	‘I am slowly coming to realise that weed is giving me anxiety and it is not making me happy. I love weed and I want to enjoy it'.	17	5.5	13	4.2	6	1.9
**TOTAL (themes)**			268	100.0	267	100.0	273	100.0
**TOTAL (sub‐themes)**			309	100.0	310	100.0	319	100.0

^a^ One tweet can have multiple themes and sub‐themes.

^b^ The percentages for the four themes (policy, sales and industry opportunities, therapeutic value, adverse effects) sum up to 100%.

^c^ The percentages for the 11 sub‐themes (e.g., support legalisation, oppose legalisation, therapeutic claims, … etc) sum up to 100%.

### 3.1 Theme 1: Policy

Tweets about ‘Policy’ were more common in illegal jurisdictions (61.5%) compared to medical only (46.8%) and fully legalised (32.5%) jurisdictions. Compared to medical‐only and fully legalised jurisdictions, Twitter users from illegal jurisdictions tweeted more frequently about ‘Support legalisation’, ‘Oppose legalisation’ and ‘Inaccessibility to medicinal cannabis’ to gather public support for legalisation. Of the tweets that provided a reason for supporting legalisation, the most common responses were it would increase patients' access to medicinal cannabis to treat chronic conditions (20.5%), reduce crime (6.9%) and create more job opportunities (2.3%) (see Table S4). ‘Other law related’ was another prominent sub‐theme that dominated the discussion across all jurisdictions and included tweets related to the need for law reforms around cannabis to reduce cannabis‐related arrests and the thriving cannabis black‐market industry in illegal jurisdictions. The sub‐theme on ‘Medicinal cannabis program’ was common only in medical‐only and fully legalised jurisdictions.

### 3.2 Theme 2: Sales and industry opportunities

‘Sales and industry opportunities’ were more common in fully legalised (26.5%) and medical‐only (19.9%) than illegal jurisdictions (12.1%). These tweets were dominated by promotional activities and vendors offering discount codes on Twitter, even in jurisdictions where cannabis was not yet legal (e.g., promoting Delta‐8 tetrahydrocannabidol (THC) products were promoted as a legal alternative to Delta‐9 THC products). Unsubstantiated claims about medical or other benefits were prevalent, particularly in fully legalised jurisdictions where 29.6% of these tweets were posted by social bots. The sub‐theme on ‘Industry‐related profits and business expansion’ that focused on investment and profits of the medicinal cannabis industry was more common in fully legalised jurisdictions.

### 3.3 Theme 3: Therapeutic value

The theme of ‘Therapeutic value’ was referenced in tweets in all jurisdictions, only slightly more often in fully legalised jurisdictions (32.1%) than medical‐only (25.1%) and illegal jurisdictions (23.8%). Tweets were largely based on public testimonials and personal experiences in using cannabis to treat chronic medical conditions. Recommendations on the type of product or dosage were also being made to other Twitter users (range = 2.3% in illegal to 6.3% in fully legalised jurisdictions). Approximately 3% to 4% of the tweets also featured research articles documenting the benefits of medicinal cannabis for a range of medical conditions or successful use of medicinal cannabis.

### 3.4 Theme 4: Adverse effects

Overall, tweets commenting on the adverse effects of medicinal cannabis use were less frequent and they were more commonly reported in fully legalised jurisdictions (9.0%) than in illegal jurisdictions (2.6%).

## 4 DISCUSSION

This study analysed the themes of 750 medicinal cannabis tweets, sampled based on a proportional sampling method that accounted for the population size of the United States. These tweets were posted between January and June 2021, a period in which legalisation referenda were passed in four US jurisdictions. The four identified themes were related to ‘Policy’, ‘Sales‐related’, ‘Therapeutic value’ and ‘Adverse effects’. Tweets were mainly posted by the public, industry, advocates and less often politicians. Very few tweets were identified as bots.

‘Policy’ was a common theme in illegal jurisdictions during the period when citizens‐initiated referenda on medicinal cannabis were debated. Our study concurs with previous study that found pro‐cannabis tweets [12, 13] particularly those advocating for public support dominated discussions on Twitter. Advocates justify their support for medicinal cannabis by expressing their dissatisfaction with the current law and lack of access to medicinal cannabis. Tweets on crime and illicit markets also featured under this theme as arguments for cannabis legalisation that highlighted criminal justice consequences and the economic benefits of legalising cannabis for non‐medicinal use, consistent with the arguments laid by those that supported cannabis legalisation [8, 9].

The other dominant theme was ‘Therapeutic value’, in which people tweeted about their own use of medicinal cannabis to treat chronic medical conditions. Recommendations were sometimes made to other Twitter users about the type of cannabis product and dosage without the direct supervision of a health professional, raising concerns about dose toxicity and potential interactions between cannabis and concomitant medications [24]. Some tweets recommended using medicinal cannabis to replace opioids for pain management, a practice reported among people who used medicinal cannabis [25]. Both practices could undermine the credibility of scientific evidence demonstrating health risks. Despite the therapeutic benefits, a small proportion of these tweets reported symptoms consistent with cannabis intoxication (e.g., feeling ‘high’) after using medicinal cannabis. It was uncertain if people know the amount of THC in the product [26], real‐time monitoring of health concerns and adverse effects on Twitter could allow us to estimate cannabis‐related harm to inform health surveillance and future research.

A substantial proportion of tweets in this study were from commercial companies and featured sales and promotion. Our findings demonstrate that the dissemination of unsubstantiated claims also occurs for medicinal cannabis on Twitter (e.g., ‘This [product] is proven to reduce anxiety’). Vendors often have large followings online and repeatedly post that could alter the public perception and social norms towards medicinal cannabis [27]. This study also found tweets posted from bot accounts often made health claims, as found in a previous study [11]. Social bots can spread misinformation and can perpetuate cannabis stereotypes which may result in a less lenient policy that could benefit the cannabis industry [28].

### 4.1 Limitations

Approximately 30% of the US tweets were excluded because the location was missing. This could underestimate the nuance in discussion. Quantitative inferences were based on 4% of the tweets sampled from the full Twitter archive nevertheless tweets were sampled in a way to ensure the representation of tweets across the United States. Discussions were largely pro‐cannabis; it is therefore likely that frequent cannabis users or interest groups such as lobbyists were overrepresented in this study. The tweets may not reflect the background context of the location where the tweets were being posted (e.g., people living in legal jurisdictions could be commenting on the laws in illegal jurisdictions). Although a comprehensive search was performed to capture medicinal cannabis tweets, it is not possible to differentiate between those who are using cannabis for medical purposes or using for medical and non‐medical purposes, highlighting the limitation of Twitter data. Additionally, only the text of the tweet was analysed. Future research could consider additional elements such as media sharing (photos and videos), news articles or conversational dynamics. During the initial coding of the tweets, the coders were not blinded to the jurisdiction type and this might influence how the themes were being coded.

## 5 CONCLUSION

This study provided a snapshot of public discourse by Twitter users about medicinal cannabis. Discussion was dominated by pro‐cannabis tweets and the key themes were broadly related to policy, therapeutic value, sales and industry opportunities, and to a less extent, adverse effects. Although our findings may not represent the views of all Twitter or cannabis users, the presence of tweets on unsubstantiated health claims, adverse effects of using medicinal cannabis, and crime‐related tweets highlighted the importance of content regulation and the continuity of monitoring public conversations to allow us to estimate cannabis‐related harms to inform health surveillance and future research.

## AUTHOR CONTRIBUTIONS

Carmen C. W. Lim: conceptualisation, methodology, formal analysis, writing—original draft preparation. Tianze Sun: methodology, formal analysis, writing—review and editing. Gary Chan, Coral Gartner, Jason Connor, Wayne Hall, Janni Leung and Daniel Stjepanovic: supervision, writing—review and editing. Janni Leung: conceptualisation, methodology, supervision, writing—review and editing. Sam Hames, Marco Fahmi: methodology, supervision, writing—review and editing.

## CONFLICT OF INTEREST STATEMENT

None to declare.

## ETHICS STATEMENT

Twitter granted academic access to publicly available historical tweets (APP ID: 21081677). Ethical clearance to analyse Twitter data was obtained from The University of Queensland's Human Research Ethics Committee (Ref: 2020/002133).

## Supporting information


**Table S1.** Search terms.
**Table S2.** Cannabis legality across US jurisdictions.
**Table S3.** Type of stakeholder or account type (*n* = 750).
**Table S4.** Reasons for supporting legalisation (*n* = 175).
