# Supplementary material for: What is the hype on #MedicinalCannabis in the United States? A content analysis of medicinal cannabis tweets
Source: Drug Alcohol Rev. 2023 Feb 21;43(1):28–35. doi: 10.1111/dar.13618 (PMC10952640; doi:10.1111/dar.13618)
Supplement: Supplementary file 1 — Table S1. Search terms. Table S2. Cannabis legality across US jurisdictions. Table S3. Type of stakeholder or account type (n = 750). Table S4. Reasons for supporting legalisation (n = 175). [file DAR-43-28-s001.docx]

**APPENDIX TABLES**

**Table S1. Search terms**

| (“medicinal marijuana” OR “medical marijuana” OR “medicinal cannabis” OR “medical cannabis” OR “medicinal weed” OR “medical weed” OR MMJ OR “medical marijuana patient”) OR ((Cannabis OR marijuana OR weed OR blunt OR stoner OR stoned OR kush OR bong OR pot OR pothead OR joint OR cbd OR cbdoil OR dabs OR 420 OR edibles OR maryjane) (migraine OR fibromyalgia OR PTSD OR insomnia OR epilepsy OR cancer OR anxiety OR “chronic pain” OR seizure)) |
| --- |

**Table S2. Cannabis legality across US jurisdictions**

| **Jurisdiction** | **Legality^a^** | **Coding** | **Size^b^** | **Sampling weight** | **Number of tweets to sample** | **Actual number of tweets** |
| --- | --- | --- | --- | --- | --- | --- |
| Alaska | Legalised | Fully legalised | 732,673 | 0.5 | 1 | 1 |
| Arizona | Legalised | Fully legalized | 7,276,316 | 5.1 | 13 | 13 |
| California | Legalised | Fully legalised | 39,237,836 | 27.5 | 69 | 69 |
| Colorado | Legalised | Fully legalised | 5,812,069 | 4.1 | 10 | 10 |
| District of Columbia | Legalised | Fully legalised | 670,050 | 0.5 | 1 | 1 |
| Illinois | Legalised | Fully legalised | 12,671,469 | 8.9 | 22 | 22 |
| Maine | Legalised | Fully legalised | 1,372,247 | 1.0 | 2 | 2 |
| Massachusetts | Legalised | Fully legalised | 6,984,723 | 4.9 | 12 | 12 |
| Michigan | Legalised | Fully legalised | 10,050,811 | 7.1 | 18 | 18 |
| Montana | Legalised | Fully legalised | 1,104,271 | 0.8 | 2 | 2 |
| Nevada | Legalised | Fully legalised | 3,143,991 | 2.2 | 6 | 6 |
| New Jersey | Legalised | Fully legalised | 9,267,130 | 6.5 | 16 | 16 |
| New York | Legalised | Fully legalised | 19,835,913 | 13.9 | 35 | 35 |
| New Mexico | Legalised | Fully legalised | 2,115,877 | 1.5 | 4 | 4 |
| Oregon | Legalised | Fully legalised | 4,246,155 | 3.0 | 7 | 7 |
| South Dakota | Legalised | Fully legalised | 895,376 | 0.6 | 2 | 2 |
| Vermont | Legalised | Fully legalised | 645,570 | 0.5 | 1 | 1 |
| Virginia | Legalised | Fully legalised | 8,642,274 | 6.1 | 15 | 15 |
| Washington | Legalised | Fully legalised | 7,738,692 | 5.4 | 14 | 14 |
| Idaho | Fully illegal | Illegal | 1,900,923 | 6.3 | 16 | 16 |
| Kansas | Fully illegal | Illegal | 2,934,582 | 9.8 | 24 | 24 |
| Nebraska | Decriminalised | Illegal | 1,963,692 | 6.5 | 16 | 16 |
| North Carolina | Decriminalised | Illegal | 10,551,162 | 35.1 | 88 | 88 |
| South Carolina | Fully illegal | Illegal | 5,190,705 | 17.2 | 43 | 43 |
| Tennessee | Fully illegal | Illegal | 6,975,218 | 23.2 | 58 | 58 |
| Wyoming | Fully illegal | Illegal | 578,803 | 1.9 | 5 | 5 |
| Alabama | Medical | Medical only | 5,039,877 | 3.2 | 8 | 8 |
| Arkansas | Medical | Medical only | 3,025,891 | 1.9 | 5 | 5 |
| Connecticut | Medical and decriminalised | Medical only | 3,605,597 | 2.3 | 6 | 6 |
| Delaware | Medical and decriminalised | Medical only | 1,003,384 | 0.6 | 2 | 2 |
| Florida | Medical | Medical only | 21,781,128 | 13.7 | 34 | 34 |
| Georgia | Medical | Medical only | 10,799,566 | 6.8 | 17 | 17 |
| Hawaii | Medical and decriminalised | Medical only | 1,441,553 | 0.9 | 2 | 2 |
| Indiana | Medical | Medical only | 6,805,985 | 4.3 | 11 | 11 |
| Iowa | Medical | Medical only | 3,193,079 | 2.0 | 5 | 5 |
| Kentucky | Medical | Medical only | 4,509,394 | 2.8 | 7 | 7 |
| Louisiana | Medical | Medical only | 4,624,047 | 2.9 | 7 | 7 |
| Maryland | Medical and decriminalised | Medical only | 6,165,129 | 3.9 | 10 | 10 |
| Minnesota | Medical and decriminalised | Medical only | 5,707,390 | 3.6 | 9 | 9 |
| Mississippi | Medical | Medical only | 2,949,965 | 1.9 | 5 | 5 |
| Missouri | Medical and decriminalised | Medical only | 6,168,187 | 3.9 | 10 | 10 |
| New Hampshire | Medical and decriminalised | Medical only | 1,388,992 | 0.9 | 2 | 2 |
| North Dakota | Medical | Medical only | 774,948 | 0.5 | 1 | 1 |
| Ohio | Medical and decriminalised | Medical only | 11,780,017 | 7.4 | 18 | 18 |
| Oklahoma | Medical | Medical only | 3,986,639 | 2.5 | 6 | 6 |
| Pennsylvania | Medical | Medical only | 12,964,056 | 8.1 | 20 | 20 |
| Rhode Island | Medical and decriminalised | Medical only | 1,095,610 | 0.7 | 2 | 2 |
| Texas | Medical | Medical only | 29,527,941 | 18.5 | 46 | 46 |
| Utah | Medical | Medical only | 3,337,975 | 2.1 | 5 | 5 |
| West Virginia | Medical | Medical only | 1,782,959 | 1.1 | 3 | 3 |
| Wisconsin | Medical | Medical only | 5,895,908 | 3.7 | 9 | 9 |

^a^ Source: Marijuana legality by state (<https://disa.com/maps/marijuana-legality-by-state>, last accessed: 20 November 2021)

^b^ Source: U.S. Census Bureau (<https://data.ers.usda.gov/reports.aspx?ID=17827>, last accessed: 1 November 2022).

**Table S3. Type of stakeholder or account type (n=750)**

| **Stakeholder/account type** | **Fully legalised (n=250)** | | **Medical only (n=250)** | | **Illegal (n=250)** | | **Total (n=750)** | |
| --- | --- | --- | --- | --- | --- | --- | --- | --- |
|  | **n** | **%** | **n** | **%** | **n** | **%** | **n** | **%** |
| General public | 117 | 46.8 | 146 | 58.4 | 130 | 52.0 | 393 | 52.4 |
| Cannabis industry^a^ | 58 | 23.2 | 37 | 14.8 | 33 | 13.2 | 128 | 17.1 |
| Cannabis advocates^b^ | 25 | 10.0 | 16 | 6.4 | 31 | 12.4 | 72 | 9.6 |
| News outlets | 22 | 8.8 | 32 | 12.8 | 19 | 7.6 | 73 | 9.7 |
| Key Opinion Leader^c^ | 9 | 3.6 | 10 | 4.0 | 20 | 8.0 | 39 | 5.2 |
| Politicians | 2 | 0.8 | 3 | 1.2 | 15 | 6.0 | 20 | 2.7 |
| Twitter bots/spam accounts^d^ | 8 | 3.2 | 1 | 0.4 | 0 | 0.0 | 9 | 1.2 |
| Unsure | 9 | 3.6 | 5 | 2.0 | 2 | 0.8 | 16 | 2.1 |

^a^Cannabis cultivators, producers, vendors, companies.

^b^Individual or organisation who publicly supports or recommends medicinal cannabis.

^c^People or organisations with a strong social status that their recommendations and opinions are listened to when making important decisions. They have a strong presence on Twitter and a large pool of followers (e.g., >5000 followers). Some examples are celebrities and columnists.

^d^These are automated or semi-automated Twitter accounts that can tweet, retweet, follow or send direct messages to other Twitter users. These bots may post rapidly and frequently using a consistent format. Bots followed a lot of accounts but may not have many followers.

**Table S4. Reasons for supporting legalisation (n=175)**

|  | *Reasons* | n | % |
| --- | --- | --- | --- |
| 1 | No reasons were cited |  |  |
|  | *Tweets are on cannabis advocacy (rallying to increase public support)* | 74 | 42.3 |
|  | *Tweets commenting on the progress on/condemning medicinal cannabis bills* | 49 | 28.0 |
| 2 | Access to medicinal cannabis to treat chronic medical conditions | 36 | 20.5 |
| 3 | To reduce crime | 12 | 6.9 |
| 4 | To create job opportunities | 4 | 2.3 |
|  |  | **175** | **100** |
